# Direct Probe Ionisation Mass Spectrometry for Rapid and Accurate Determination of Perfluoroalkyl and Polyfluoroalkyl Substance Concentrations

**DOI:** 10.1002/rcm.10079

**Published:** 2025-06-11

**Authors:** Tim P. Sidnell, Simone C. Mathias, David P. Megson, Patrick J. Sears, Madeleine J. Bussemaker

**Affiliations:** ^1^ School of Chemistry and Chemical Engineering University of Surrey Guildford UK; ^2^ Ecology & Environment Research Centre Manchester Metropolitan University Manchester UK

**Keywords:** ambient ionisation, mass spectrometry, perfluoroalkyl substances, PESI‐MS, PFAS

## Abstract

**Rationale:**

Analysis of perfluoroalkyl and polyfluoroalkyl substances (PFAS) is typically achieved using chromatographic techniques, such as high‐performance liquid chromatography, combined with mass spectrometry. These techniques can be complex, expensive, time consuming and can lead to inaccurate quantification due to sample container sorption and contamination by polytetrafluoroethylene (PTFE) components.

**Methods:**

Here, we demonstrate the novel application of a Direct Probe ionisation Mass Spectrometer (DPiMS) to quantify four aqueous anionic PFAS; perfluorooctanoic acid (PFOA), perfluorooctane sulfonic acid (PFOS), perfluorononanoic acid (PFNA) and 1H,1H,2H,2H‐perfluorooctanesulfonic acid (6:2 fluorotelomer surfactant). The DPiMS was operated in both Single Ion Monitoring (SIM) and broad *m/z* scan range (50–500) modes and the cleaning methodology, ionising voltage and desolvation line heating temperature were optimised.

**Results:**

The derived method quantified both single component and mixtures of PFAS from 0.5 nM to 200 μM (0.2 ppb to 108 ppm) without use of pre‐concentration. The advantages of this technique over chromatographic techniques are; the speed of analysis (≈3.5 min per sample, including blanks/cleaning), lack of PTFE components and simplified methodology.

**Conclusions:**

The DPiMS is a useful tool for the rapid screening and estimation of PFAS concentrations with results that are comparable to existing methods. It is anticipated that better sensitivity could be achieved with the use of a triple quadrupole instrument.

## 1 Introduction

Perfluoroalkyl and polyfluoroalkyl substances (PFAS) are chemical compounds containing the perfluoroalkyl moiety (C_n_F_2n + 1_) [1], which have been in mass production and use since the 1940s, in products, including grease‐proof packaging [2], aqueous fire‐fighting foams (AFFFs) [3], waterproof textiles, ski waxes [4], non‐stick cookware [5]. However, as knowledge of their effects on the living world has grown [6, 7], there have been increasing concerns over PFAS production, use and disposal [8, 9]. Their bio‐accumulative properties [10] and link to long term health effects such as endocrine disruption [11], infertility [12] and cancer [13, 14] are among chief concerns which led to the two previously most used PFAS (perfluorooctanoic acid and perfluorooctane sulfonic acid, PFOA/S) and related compounds to be controlled under the Stockholm Convention as persistent organic pollutants (POPs) [15, 16]. PFAS pollution is typically greatest in emissions from PFAS manufacturing facilities [17], landfill leachate from consumer products and construction materials [18] and AFFFs used in military and aviation fire training [3]. However, PFAS are water soluble and easily spread into soil, rivers, plants, animals, oceans and wastewater treatment plants (WWTPs), leading to sparse, yet ubiquitous, prevalence throughout the hydrosphere [17, 19].

Groundwater directly impacted by AFFFs may contain several mg L^−1^ of PFAS [3, 20], while WWTP effluent can contain several μg L^−1^ [21], rivers and estuaries a few ng L^−1^ [17, 22] and oceans just pg L^−1^ [17, 19]. The UK Drinking Water Inspectorate has recommended that individual PFAS in water should not be greater than 100 ng/L, however there are currently no legal standards for PFAS in drinking water within England and Wales or a World Health Organisiation guideline value [23]. It has been estimated that at least 4730 PFAS exist, therefore accounting for total PFAS concentrations is challenging [24]. This is further convoluted by the complexity of environmental samples, such as living tissue [22], AFFFs [3] and groundwater, which may can contain several PFAS, co‐organics, inorganics and solvents, which mask or enhance PFAS detection [20]. Hence, there is a need to qualify and quantify a plethora of aqueous PFAS, in numerous samples types and across concentrations spanning many orders of magnitude. PFAS analysis is typically completed using (ultra) high performance liquid chromatography [(U)HPLC] [9, 25] coupled with a choice of mass spectrometer (MS), mostly governed by selectivity and limits of detection/quantification (LOD/Q) [26, 27]. For example, an orbitrap detector was able to quantify perfluorodecanoic acid (PFDA) at just 0.03 ng L^−1^ [28].

PFAS analysis using (U)HPLC‐MS typically requires a solvent gradient function which takes 8–9 min to complete [22, 29, 30], or up to an hour for isomeric separation [31]. After column flushing and blanks, these techniques can only analyse one to three samples per hour [31]. Such analysis times affect the measured concentration, since PFAS irreversibly sorb sample container materials over time. Polypropylene, polystyrene, polycarbonate and glass centrifuge tubes absorbed approximately 40%, 35%, 30% and 20%, respectively, of 21.32 μg L^−1 14^C‐PFOA over 24 h [32]. Similar results were seen for several PFAS using nylon, polytetrafluoroethylene, and polyethersulfone filters [33]. Hence, rapid analysis is critical to minimise sample loss in glass chromatography autosamplers vials. The column, elution solvents and mobile phase gradients must also be carefully selected for sufficient separation of multiple PFAS [20]. These factors represent further financial and temporal costs and limit application to those with significant analytical chemistry experience. Chromatography can also artificially enhance some PFAS concentrations, due to polytetrafluoroethylene (PTFE) coatings in tubing [9], sample caps [34] and other parts of the system, which must be replaced with stainless steel or polyetheretherketone alternatives [9, 35]. In one study, PTFE‐lined tubing contributed to PFOA, perfluorononanoic acid (PFNA), perfluorodecanoic acid (PFDA) and perfluoro‐undecanoic acid (PFUnA) contamination within a UHPLC–MS/MS. When analysing 0.1 μg L^−1^ PFOA, the PTFE contributed ≈70% of the PFOA intensity [35]. Such effects would be significant when analysing PFAS in WWTPs [21] and would render analysis of river and oceanic waters [17, 22] invalid.

Alternative analytical technologies exist, which overcome some of these concerns. For example, total oxidizable precursor (TOP) assay reduces the number of assessable PFAS, by oxidising perfluorocarboxylic acid (PFCA) precursors (polyfluorinated PFAS which degrade into perfluorinated PFAS) into PFCAs [36]. However, this masks sample understanding and adds greater complexity, cost and time to analysis, without resolving the issues with chromatography. TOP assay is also less suited to short chain (≤C4) precursors, due to signal suppression by high post‐oxidation salt concentrations [37]. Ion selective electrodes [38], particle induced gamma ray emission (PIGE) spectroscopy [39, 40] combustion ion chromatography (CIC) and instrumental neutron activation analysis (INAA) are useful for quantifying fluorine as a proxy for total PFAS but not individual PFAS concentrations [41]. A select few non‐chromatographic methods exist for individual PFAS analysis. For example, high‐field asymmetric ion mobility spectrometry (FAIMS)‐MS, uses isomeric transport property differences to rapidly separate PFAS isomers (within 3 min, excluding blanks) [31]. However, FAIMS‐MS is not yet proven for non‐isomeric (multi‐PFAS) separation and is yet to be considered across several environmentally relevant concentrations [31]. Aggregated fluorescence uses aggregation‐induced emission luminogens (AIEgens), to emit a PFAS concentration dependant level of fluorescence. This gives quantification within just 1 min, but over a limited concentration range (0.1–100 μM) [42]. Nuclear magnetic resonance (NMR) has also been investigated for aqueous PFAS quantification [43], although it is still experimental and requires comparatively larger, more complex and expensive equipment than chromatography.

There is, a need to develop novel analytical methods which hasten, simplify and cheapen PFAS analysis, with limited compromise on quantification or qualification sensitivity. The direct probe ionisation mass spectrometer (DPiMS) is an ambient temperature and pressure mass spectrometry technology, and is a commercial version of the technique probe electrospray ionisation (PESI) developed by K. Hiraoka et al. in 2007 [44], which does not require a column, solvent gradient or PTFE components. PESI utilises a solid needle which moves up and down. The needle is lowered into a sample and then raised in line with the mass spectrometer inlet, at which point a high voltage (typically 2–3 kV) is applied forming an electrospray at the tip of the needle [45]. The analyte ions produced then enter the inlet of the mass spectrometer for analysis. Ambient ionisation mass spectrometry is considered simpler [46], quicker and more cost effective [47] than high pressure techniques, but perhaps less sensitive. Ambient ionisation mass spectrometry is becoming more recognised for PFAS analysis, for example, solid phase microextraction coupled with techniques such as direct analysis in real time [48], wooden tip probe spray [49] and metal needles for a form of PESI [50, 51]. To the best of the authors' knowledge, this manuscript describes the first DPiMS PESI method for rapid and accurate determination of aqueous PFAS. The results describe the following aims and objectives in developing this methodology. Aim: To establish if DPiMS is a useful and rapid tool to determine aqueous PFAS concentrations. Objectives: (1) To establish suitability of the method in scan mode for multi‐PFAS screening, (2) to optimise method performance for specific PFAS and (3) to demonstrate optimised method effectiveness for rapid low‐level analysis of multiple specific PFAS.

## 2 Methodology Development

### 2.1 Materials

The ≥99.8% HPLC grade acetone, 95% perfluorooctanoic acid (PFOA) [CF_3_(CF_2_)_6_COOH], ≥98.0% potassium salt of perfluorooctane sulfonic acid (K‐PFOS) [CF_3_(CF_2_)_7_SO_3_K], 98% 6,2‐fluorotelomer surfactant (6:2FTS) [CF_3_(CF_2_)_5_(CH_2_)_2_SO_3_H] and 97% perfluorononanoic acid (PFNA) [CF_3_(CF_2_)_7_COOH] were purchased from Merck. LCMS Optima grade methanol and ≥99.8% analytical grade ethanol were purchased from Fisher Chemical. Ultrapure water was provided by an in‐house Milli‐Q device. Samples were analysed using a Shimadzu Ltd. Direct Probe ionisation Mass Spectrometer (Model: DPiMS‐2020, a single quadrupole mass spectrometer).

### 2.2 Operational Modes and Signal Analysis

Using the DPiMS, broad *m/z* scan range and single ion monitoring (SIM) modes were investigated, and the mass spectrometer was operated in negative mode, since all PFAS assessed are anionic. The sample probe performed ≈40 extractions per minute (Figure S1) and the total ion chromatogram (TIC) shows corresponding fluctuations in intensity (arbitrary units, AU) (Figure S2). The signal was integrated using the associated LabSolutions software to give an average intensity during sampling times 0.1–1.0 min, which excluded the peak containing significant noise at 0.0–0.1 min (Figure S2). Further details on DPiMS operation are described in Section S1.

Several parameters affect the DPiMS intensity reading, the most significant of which were identified from the DPiMS‐2020 manual [52] and the authors' experience of the device. These factors were then assessed and optimised on a ‘one factor at a time basis’ to achieve the highest intensity peak, as used previously [28]. The order of parameters optimised and assessment ranges were; sample concentration (0.5 nM to 200 μM [0.2 ppb to 108 ppm]), monitoring mode (Scan/SIM), sample slide cleaning (Yes/No), probe cleaning (Yes/No), desolvation line temperature (100°C–250°C) and ionisation voltage (−2.45 to −1.75 kV). Justification of parameters and optimisation order is given in the supplementary information, Section S3 and Table S2.

### 2.3 Calibration Series

Performance of the DPiMS was assessed using two dilution series of PFAS powders dissolved in ultrapure water. Series 1, established DPiMS suitability for multi‐PFAS screening and was composed of three sub‐series (1.1, 1.2 and 1.3), containing PFOA, PFNA and PFOS, respectively, at concentrations of 0.002–200 μM (0.8 ppb to 108 ppm). Series 2 assessed the matrix effects of four combined PFAS (PFOA, 6:2 FTS, PFNA and PFOS) with the same total PFAS concentration as series 1.1–1.3 and 25% of the individual PFAS concentrations (50–0.0005 μM [21 ppm to 0.21 ppb]). Since engineers, chemists and environmental scientists favour different concentration metrics, Table S1 shows the relative molecular masses (RMMs) in Daltons (Da) of the four PFAS tested and their concentrations in each series in micromoles per litre (μM), milligrammes per litre (mg L^−1^) and parts per billion (PPB). Further details on dilution series manufacture are given in Section S2.

### 2.4 Background Subtraction and Limits of Detection/Quantification

Blank samples containing a 1:1 v/v ratio of Milli‐Q water and ethanol were used to assess the background noise intensity prior to each analyte sample, which was subtracted from the subsequent sample's intensity. The limit of detection was defined as a signal detected at three times the intensity of a blank signal. Where an internal standard was used, the blank comprised a 1:1:2 v/v ratio of Milli‐Q water:aqueous internal standard:ethanol, replicating the 1:1 ratio of aqueous and organic phases in all samples and blanks. No background subtraction was completed for the internal standard since the same concentration was used in both blanks and samples.

### 2.5 Cleaning Regime

Initial investigations revealed sample carryover, since successive repeats of high concentration samples showed increasing intensity. Therefore, a cleaning sample containing 10 μL acetone was run before each blank, to remove any residual sample on the needle and in the desolvation line. The intensity response to acetone was not beyond background noise and hence did not effectively ionise the residual sample. Between samples, blank and analyte slides were dismantled and washed with 3 × 1 mL ≥ 99.9% methanol, using a polypropylene pipette tip, to remove any sample residue. The slides were then blotted with lint free tissue and left in a fume cupboard to evaporate residual methanol. To further minimise carryover, the analyte, cleaning and blank slides were not interchanged. To avoid possible masking effects, the internal standard was kept at the mid‐concentration (2 μM [0.9 ppm]) of the detectable logarithmic range (2 nM to 200 μM [0.8 ppb to 108 ppm]), as per prior work [9, 22]. The calibration series were sampled in ascending concentration to minimise relative carryover. However, between series, the last sample of the prior series would be significantly more concentrated (50–200 μM [21–108 ppm]) then the first of the following series (0.5–2 nM [0.21–1.1 ppb]). Therefore, several blanks (typically ≈4) were run at the start of each series to remove any residue in the MS. Once a week, the desolvation line was placed into maintenance heating mode to remove any sample remaining in the device at ~350°C. The desolvation line was then detached and flushed with 20 mL water, methanol then acetone, to remove any remaining residue.

### 2.6 Internal Standard Selection

Initial experiments were performed on separate solutions of PFOA, PFNA and PFOS. As a proof of concept for quantitative analysis additional experiments were then run which quantified PFOA by using PFNA as an internal standard. PFNA was selected as the internal standard for PFOA rather than PFOS, as it was from the same series (C9 vs. C8 PFCA) and therefore has a similar anionic nature and [M‐H]^−^ value close to PFOA, (*m/z* of 463 vs. 413) PFAS manufacturing processes can, however, produce several PFAS with a range of chain lengths and degrees of branching [53, 54]. Therefore, a broad scan range analysis was conducted to confirm PFNA presence in the highest PFOA solution (200 μM [83 ppm]) was indistinguishable to PFNA levels in the blanks (Figure S3). Further discussion on the choice of internal standard is given in the Section S4.

## 3 Results and Discussion

Three separate experiments were performed to investigate the three main objectives of this research: (1) Individual solutions of PFOA, PFNA and PFOS were analysed separately in full scan mode. Decreasing concentrations of each solution were analysed to estimate the linear range and limits of detection to establish the suitability of the method for multi‐PFAS screening in full scan mode. (2) The settings on the DPiMS probe were optimised for PFOS and selected ion monitoring used to demonstrate how performance can be modified to target one specific PFAS, and (3) calibration curves were prepared to demonstrate the optimised method effectiveness for rapid low‐level analysis of multiple specific PFAS through investigation of the upper and lower limits of quantification based on the linear range.

### 3.1 Establishing the Suitability of DiPMS to Measure PFAS in Full Scan Mode

**FIGURE 1:**
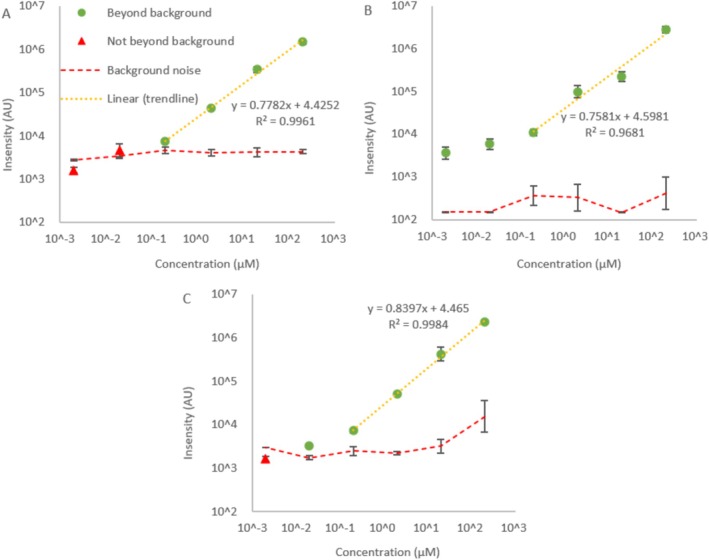
Signal intensity in arbitrary units (AU) versus PFAS concentration (μM): PFOA (A), PFOS (B) and PFNA (C). Intensities beyond background noise in two out of three repeats (green circles) and not beyond background noise (red triangles) as well as the background noise at each concentration (red dashed line) and linear region of quantification (yellow dotted line) during DPiMS analysis using a broad scan range of *m/z* 50–550. Error bars represent the standard deviation of three repeats.

A non‐targeted broad scan range analysis was applied over the mass range *m/z* 50–550 using concentrations of PFOA, PFNA and PFOS covering the range 0.002–200 μM (0.8 ppb—108 ppm) (Series 1.1–1.3 in Table S1). The samples contained a 1:1:2 v/v ratio of aqueous PFAS: water: ethanol and were made and tested in triplicate. The ionising voltage, desolvation line heating temperature, probe immersion depth and ionising depth were −2.45 kV, 250°C, −45.5 mm and −37 mm, respectively. PFOA intensity in both the samples and blanks was measured at a *m/*z 413, representing the [M‐H]^−^ ion. The DPiMS detected PFOA across a concentration range of 0.2–200 μM (83 ppb to 83 ppm) while readings at 2–20 nM (0.83–8.3 ppb) were not consistently detected above background noise (Figure 1A). Data in Figure 1 are not blank subtracted and the background noise at each concentration is shown. Samples of PFOS and PFNA were analysed at *m/z* 499 and 463, respectively, and show similar trends in intensity versus PFAS concentration (Figure 1B,C).

Linearity was achieved over at least three orders of magnitude PFOA concentration (0.2–200 μM [83 ppb to 83 ppm]) using the trendline *y* = 0.7782*x* + 4.4252 (*R* ^2^ = 0.9961), where y = log_10_(intensity) (AU) and *x* = log_10_[PFOA] (μM). Excluding the data at 200 μM (83 ppm) from the trendline gives an increased *R* ^2^ of 0.9976 (*y* = 0.8287*x* + 4.4269), while including data at 0.02 μM (83 ppb) reduces the *R* ^2^ to 0.9700 (*y* = 0.6662*x* + 4.5710). Hence, there is some deviation from linearity above 20 μM (8.3 ppm) and more extreme deviation below 0.2 μM (83 ppb). Extrapolating *y* = 0.7782*x* + 4.4252 beyond 200 nM (83 ppb) predicts the same intensity as the background noise (≈3900 AU) at 98.04 nM (41 ppb), thus suggesting the lower LOQ. The slopes and constants of the linear regions in Figure 1B,C, PFOS and PFNA, respectively, are similar to those of PFOA, suggesting a comparable response of the MS. However, for PFOS, the LOD was two orders of magnitude lower than for PFOA (2 nM (1.1 ppb)) and linearity was harder to assess due to an anomalously low intensity at 20 μM (11 ppm). An approximate linear region is suggested in Figure 1B. Due to overlap between the standard deviations of intensity at 2 (1.1 ppb) and 20 nM (11 ppb), the lower limit of linear response to PFOS should occur between 20 and 200 nM (11–108 ppm) at ≈4190 AU. Extrapolating the trendline to this intensity reveals the lower LOQ to be 99.1 nM (54 ppb), near identical to that of PFOA. The LOD of PFNA was lower than for PFOA but higher than PFOS, at 20 nM (9.3 ppb) (Figure 1C). The linear region between 0.2 and 20 μM (93 ppb—9.3 ppm) fits the equation *y* = 0.8397*x* + 4.465 (*R* ^2^ = 0.9984). Extrapolation reveals the lower LOQ to be 82.4 nM (38 ppb) (at ≈1600 AU). Thus, linearity was achieved over at least three orders of magnitude for all three PFAS.

The results show that the DPiMS method has potential as a rapid screening tool for PFAS as it was able to detect PFOA, PFNA and PFOS in a simple sample. Linear ranges of approximately 3 orders of magnitude were observed for each PFAS with detection limits of 2–20 nM (0.8–11 ppb) depending on the PFAS being analysed. PFCAs and PFSAs are two of the most commonly analysed PFAS and form the basis for many routine monitoring and remediation programmes, which would benefit from a rapid analytical method [55, 56]. However, due to the complexity of PFAS chemistries we would recommend testing on further PFAS classes to establish how effective this method may prove as a wider PFAS screening tool.

### 3.2 Targeted SIM Analysis

#### 3.2.1 Parametric Optimisation for Improved PFOA Detection

Increasing device sensitivity, by lowering the LOD, and extending the linear response range were the chief objectives when optimising the instrument parameters during SIM mode. PFOA was selected for this optimisation, since it had the highest LOD in the initial study (Section 3.1). Parametric effects were assessed using a 1:1:2 v/v ratio of 2 μM (0.8 ppm) PFOA:2 μM (0.9 ppm) PFNA:ethanol.

##### 3.2.1.1 Cleaning Regime

Investigations revealed sample carryover was occurring at high PFAS concentrations (Figure 1B,C). PFAS have a sticky and surfactant type nature [8, 32, 33] and this carryover appeared to be due to residue on the sample slide, probe or desolvation line which contaminated proceeding samples. Experiments with previously unused sample slides revealed that the majority of carryover occurred within the slides. The slide cleaning regime described in Section 2. was implemented and took less than 1 min, meaning that it could be completed while another sample was running. The remainder of the carryover, within the DPiMS itself, was mitigated using an acetone blank, ensuring reliable and consistent readings. This methodology likely also improved the LOD and LOQ, by reducing the background intensity to be subtracted from subsequent samples.

##### 3.2.1.2 Ionising Voltage Desolvation Line Temperature

**FIGURE 2:**
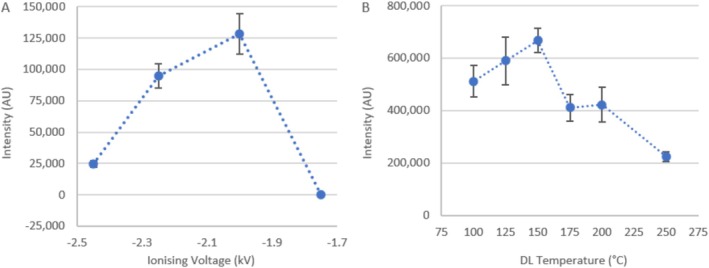
Intensity in arbitrary units (AU) of 2‐μM PFOA under SIM mode using ionising voltages of −2.45 to −1.75 kV and desolvation line heating temperature of 250°C (A) and using desolvation line heating temperatures of 100°C–250°C and ionising voltage of −2.00 kV (B). Error bars represent the standard deviation of three repeats.

The DPiMS ionising voltage is modifiable from −0.5 to −3 kV (during negative mode operation). At a desolvation line heating temperature of 250°C, the ionising voltage was varied from −2.45 to −1.75 kV and peak intensity was achieved at −2.00 kV, giving ~5× the initial intensity at −2.45 kV (128 000 vs. 24 700 AU) (Figure 2A). Then, holding the ionising voltage at −2.00 kV, desolvation line heating temperature was varied from 250°C to 100°C. The highest peak intensity was achieved at 150°C, with ~3× that seen at the initial 250°C; however, there was statistical overlap between the repeats at 150°C and 125°C (Figure 2B).

The ionising voltage applied to the probe/sample determines the volume of analyte observed. Under too high a voltage, analyte ions will be fragmented and hence not detected at the anticipated *m/z* values. However, too low a voltage will not ionise the sample or overcome the Rayleigh limit to form an electrospray (Taylor cone) [46, 57]. Figure 2A shows that the minimum (or onset) [46] voltage at which PFOA forms an ionised electrospray is between −2.00 and −1.75 kV. Fragmentation of PFOA appears to increase as the absolute voltage is increased in magnitude beyond −2.00 kV. Ionising voltage also affects the shape of the Taylor cone emitted from the probe during ESI, with; dripping, spindle, pulsed cone, stable cone and multi‐jet sprays being observed from low to high (magnitude) voltages [58]. Hence, the reduced intensity at increasingly negative voltages may also arise due to high magnitude voltage spray regimes, which are known to permit less droplets to reach the MS [58].

The desolvation line temperature controls the mass of solvent evaporated from the electrospray droplets. Figure 2B shows that low temperatures (100°C) do not evaporate enough solvent to achieve optimal PFAS detection. The lower intensity seen at higher temperatures (175°C–250°C) conversely suggests that too much solvent was evaporated, which limited droplets in carrying PFOA^−^ to the detector, since long chain perfluorinated PFAS are non‐volatile [17]. It is also possible that the solvent may have evaporated too rapdily at these temperatures, leading to inefficient ionisation or thermal degradation of the PFAS. The desolvation temperatures used in the detection of PFAS using traditional electrospray ionisation methods are typically much higher (350°C–550°C) [4, 59, 60, 61, 62] than the optimised values presented in Figure 2A,B. This may be due to the greater liquid volume injection into the ESI source in LC, compared with the droplets formed with PESI, which will require greater temperatures to evaporate the excess solvent and which otherwise lead to ion cluster formation [46].

#### 3.2.2 PFOA Quantification Using Optimised Parameters

**FIGURE 3:**
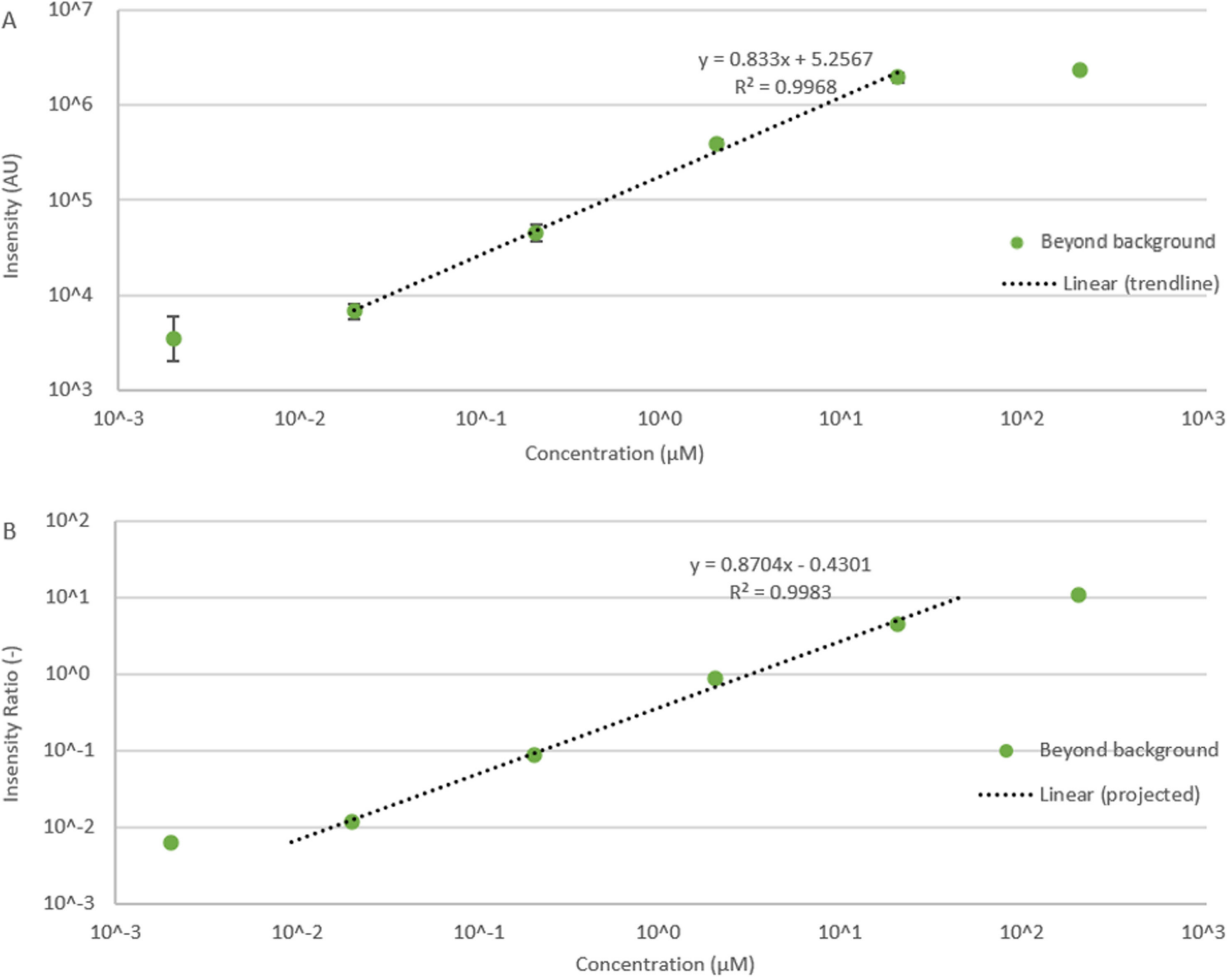
Background subtracted DPiMS intensity in arbitrary units (AU) versus PFOS concentration (μM) (A) and background subtracted DPiMS intensity ratio (dimensionless) (B) of PFOA:PFNA versus PFOS concentration (μM) for PFOA of concentrations 2 nM to 200 μM using optimised ionising voltage and desolvation line heating temperature. Error bars (not always visible) represent the standard deviation of three repeats.

Using the optimum ionising voltage and desolvation line temperature (Section 3.2.1), the calibration curve for PFOA was re‐measured (Figure 3A, background subtracted). The LOD was improved by 2 orders of magnitude (2 nM [0.8 ppb]) compared to the results in Section 3.1. The calibration curve using the ratio of PFOA:PFNA intensities is shown in Figure 3B and the linear trendline for log_10_(intensity PFOA/intensity PFNA) versus log_10_[PFOA] covers concentrations from 20 to 0.02 μM (8 ppm to 8 ppb). The upper and lower linear intensity limits shown in Figure 3A are 2.30 × 10 [6] and 3500 AU, respectively, similar to those under scan mode (1.46 × 10 [6] and 3900 AU). However, extending the trendline in Figure 3B to these limits gives the upper and lower LOQs as 47.0 μM (19 ppm) and 9.22 nM (4 ppb), respectively. Thus, the linearly quantifiable PFOA concentrations cover almost 4 orders of magnitude, compared with 3 orders of magnitude under SIM mode.

The results of this experiment show how the DPiMS method can be optimised for use for one specific PFAS. In a simple solution a linear range of 4 orders of magnitude were observed for each PFOS with detection limits of 2 nM (0.8 ppb). This represented a significant improvement over the full scan method and demonstrates how this method has potential to be used for rapid targeted analysis of a specific PFAS.

### 3.3 Method Validation via Targeted Multi‐PFAS SIM

**FIGURE 4:**
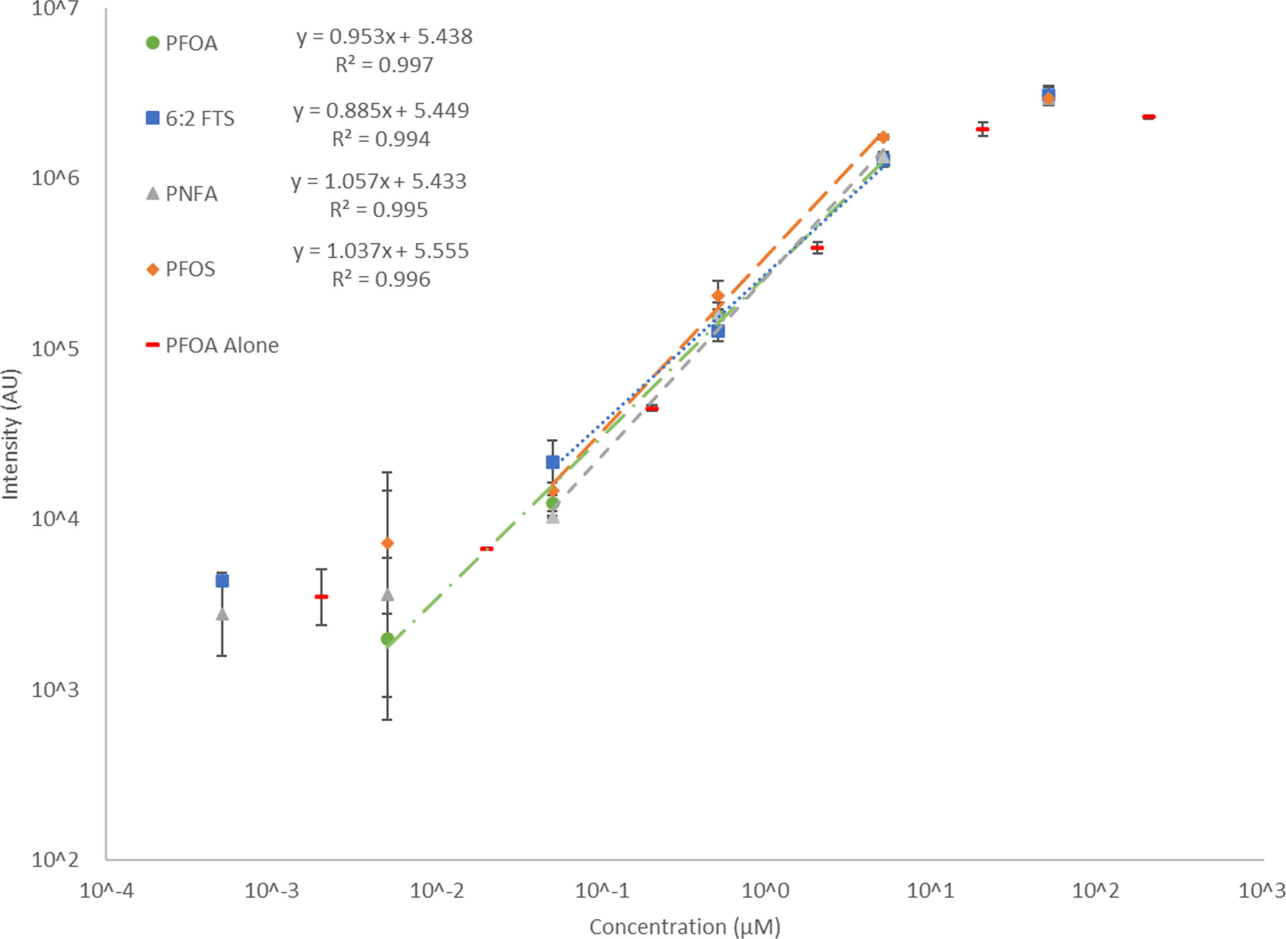
Plot of intensity in arbitrary units (AU) for the individual PFAS in the four‐PFAS samples, under the optimised ionising voltage and desolvation line temperatures. Error bars represent the standard deviation of three repeats.

A solution containing four PFAS (Series 2) was used to observe any potential competitive ion‐source ion suppression effect between PFAS and assess the optimised method's suitability for analysing multiple PFAS. The four PFAS (PFOA, PFNA, 6:2 FTS and PFOS) were analysed using *m/z* values of 413 [M‐H]^−^, 463 [M‐H]^−^, 427 [M‐H]^−^ and 499 [M‐K]^−^, respectively. Samples contained 0.5 nM to 50 μM (0.2 ppb to 27 ppm) each of four anionic PFAS and thus represented the same total PFAS concentrations in series 1. All four PFAS showed the same characteristic sigmoidal intensity versus concentration plot and similar intensity levels (within 1 order of magnitude) at any given concentration (Figure 4). All PFAS had good linearity between 0.5 and 50 μM (0.2–27 ppm) (0.05 μM [2 ppb] for PFOA) with coefficient of determination values (*R* ^2^) of >0.994 and an average slope of 0.9825 (±10.2%) and intercept of 5.4685 (±0.7%). This suggests that other anionic PFAS should behave similarly within the device at moderate to high concentrations, showing a predictable increase in intensity with concentration.

The results suggest that differences in structure including, acid groups (sulphonic acid PFOS and 6:2 FTS versus carboxylic PFOA and PFNA), degrees of fluorination (polyfluorinated 6:2 FTS vs. perfluorinated PFOS, PFOA and PFNA) and chain lengths (C8 PFOA, PFOS and 6:2 FTS vs. C9 PFNA) had little impact in DPiMS intensity response [33]. Figure 4 demonstrates similar intensity responses for pure and multi‐component PFOA. The small increase in PFOA intensity in the multi‐PFAS mixture indicates positive matrix effects for signal enhancement, as observed in prior PFAS research [9].

The physical explanation for the sigmoidal trend of intensity versus PFAS concentration appears threefold. Firstly, at concentrations below and approaching ≈50 nM (≈20 ppb), the low ion count was not detectable against background noise, leading to the plateau at ≈3500 AU. Secondly, at mid‐range concentrations the intensity is linearly proportional to the count of PFAS ions reaching the MS detector. Thirdly, at concentrations approaching and above 50 μM (≈20 ppm), the intensity is saturated by the MS upper detection limit [52], hence the diminishing intensity enhancement with increasing concentration. Table 1 shows the LOQs for the PFAS tested, derived by extrapolating the linear trendlines of Figure 4 to the apparent limits of intensity, as well as the LODs. The LOQ for 6:2 FTS is assumed since the data at 50 nM was too noisy. Table S3 shows the LODs and LLOQs in alternative units.

**TABLE 1:** LOD and LOQs for the four PFAS tested in the multi‐PFAS samples under optimised conditions in μM and ppb.

PFAS	LOD		Linear range of quantification (lower and upper LOQs)	
	μM	ppb	μM	ppb
PFOA	2.00 × 10^−3^	0.828	0.0057	2.36
			−12.55	−5200
6:2 FTS	5.00 × 10^−4^	0.214	*0.0159	6.81
			−15.8	−6770
PFNA	5.00 × 10^−4^	0.232	0.0170	7.89
			−9.62	−4460
K‐PFOS	5.00 × 10^−3^	2.69	0.0233	12.5
			−7.66	−4120

* Assumed.

### 3.4 Implications for PFAS Detection and Environmental Remediation

PFAS contaminate the environment at concentrations ranging from pg m^−3^ in air and oceans, to several μg kg^−1^ in soil/sediment and μg L^−1^ in groundwater, landfill leachate and WWTP effluent [17, 20]. The methodology presented here demonstrates the ability of a single‐quad DPiMS to analyse anionic, aqueous PFAS down to 214 ng L^−1^, which therefore has wide applicability at several commonly encountered environmental concentrations. The United States, which has one of the strictest PFAS drinking water limits worldwide, as low as 4 ng L^−1^ for PFOA and PFOS concentrations [63] would require an improved LOD which may be possible with the use of more a sensitive mass spectrometer, such as a quadrupole time of flight (Q‐TOF), orbitrap [28] or triple quadrupole (now available for this technique). In this work we typically observed LODs of around 1–3 ppb (depending on the PFAS) and could expect at least an order of magnitude improvement in sensitivity using a triple quadrupole instrument due to the improvement in signal‐to‐noise ratio.

A limitation of using quadrupole mass spectrometers is not being able to differentiate between isobaric compounds due to the low resolution of the instrument. It may be possible to induce ‘in‐source’ fragmentation by altering the cone voltage, as demonstrated in previously published research [64, 65], which could help with identifying isobaric compounds using the fragmentation pattern. A triple quadrupole mass spectrometer may help with the separation of isobaric compounds based on the observed fragmentation pattern.

While the most accurate concentration measurements are achieved using isotopologues as internal standards, these are not available for all the 4730+ environmentally detectable PFAS [24]. The responses of the DPiMS to PFOA and PFNA are within ±4.5% from 0.5–50 μM (0.2–23 ppm) and within ±20% at 0.05 μM (≈20 ppb), with similarity decreasing at lower concentrations. Further, PFOA shows an intensity within ±26.4% of PFOS's from 50–0.05 μM (27 ppm to 27 ppb). The results show that the DPiMS is suited for use as an initial screening tool, providing semi‐quantitative analysis as order‐of‐magnitude concentration estimations are possible for species without suitable analytical internal standards when using similarly structured and similarly charged analogues under this methodology. Quantification is possible using the linear response of the calibration curves although the inclusion of an isotopologue would improve the accuracy of the results, or alternatively samples could be submitted for traditional analysis by LC–MS. The comparable intensity values for single‐ and multi‐PFAS PFOA suggests that use of multiple PFAS can hasten calibration curve development and thus speed of analysis while still closely reflecting the intensity response of unknown samples, which may themselves be pure or, more likely, multi component mixtures [17, 53, 54].

At the inflection points of the PFOA trendlines (≈0.5–2 nM [0.2–0.8 ppb] and 50–200 μM [21–83 ppm]) the pure PFOA trendline deviates from that of the multi‐component mixture. This is likely because the two were taken with differing concentration intervals, thus, giving a lack of resolution over the straight‐line sections of the inflection points. Future work would, therefore, benefit from an increased number of concentrations investigated about these points. Analysis close to or above 200 μM (83 ppm) will allow detection but not accurate quantification, since large variations in concentration will lead to minor intensity changes, indistinguishable from intrinsic measurement fluctuations. Therefore, where initial tests indicate a concentration above ≈10 μM (≈4 ppm), the sample should be diluted and re‐analysed until the device indicates a concentration within the LOQs. Similarly, solid phase extraction should be used to increase concentrations ≤0.2 μM (100 ppb) [28, 43, 59].

**FIGURE 5:**
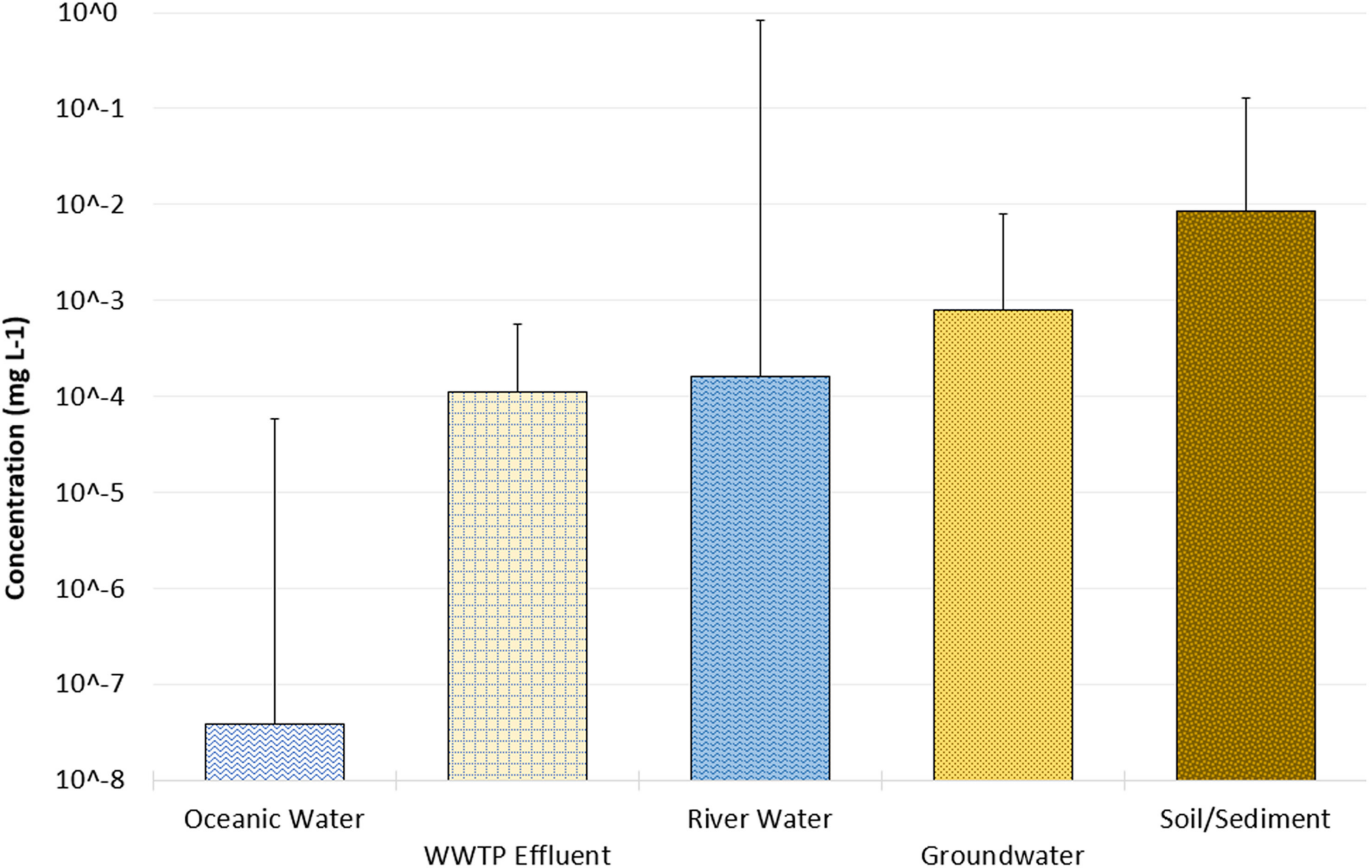
Typical PFAS concentrations (bars) and upper limit detected [66] in five environmental sample*.*

Figure 5 shows typical environmental PFAS concentrations in a range of scenarios to enable comparison to analytical technology. From literature review liquid chromatography mass spectrometry is the most described technique for the analysis of PFAS which when partnered with a preconcentrating sample preparation technique can enable limits of detection in the low parts per trillion (ppt) [20, 22, 35, 67]. The drawbacks of these techniques being the large volumes of solvent needed and the long time for both sample preparation and analysis.

Although in this study DPiMS was not as sensitive as LC–MS the linear dynamic range is comparable to (or better than) many of these studies. The use of a triple quadrupole or high‐resolution mass spectrometer could make DPiMS more appropriate for low level contamination analysis as could the use of a preconcentration technique (e.g., SPE) which has been shown to increase system sensitivity by 2 orders of magnitude [17, 22, 35].

When comparing DPiMS to techniques more suited to rapid analysis (such as ion mobility spectrometry, laser diode thermal desorption [28] or fluorescence spectrometry [42]) the time taken for the analysis is comparable (in the order of minutes). PFAS treatment technologies typically operate at higher concentrations than environmental matrices. Several works have assessed ultrasonic PFAS breakdown at ≈1–10 mg L^−1^, since this concentration optimises reaction rates [68], as seen for electrochemical oxidation [69], plasma treatment [70] and several other technologies [71, 72, 73]. DPiMS is ideal for hastening analysis of PFAS treatment technologies and could confirm up to ≈99.998% destruction (0.2 μg L^−1^ of 10 mg L^−1^). The DPiMS could also analyse non‐destructive PFAS removal technologies, such as sorption to granular activated carbon, which typically concentrate PFAS to several μg L^−1^ [74].

The novelty of this work comes from the simplified methodology, which is easily applied by those with little analytical chemistry background, since choice of columns, ramping functions and elution solvents are not required. The authors were able to manually analyse ≈15 PFAS samples per hour (45 in total, including blanks and cleaning runs) which might be considered competitive with more established auto‐sampling devices, usually run overnight or while completing other work. To the best of the authors knowledge, this is the first example of PESI‐MS being used for the analysis of PFAS and demonstrates a starting point for future researchers. Future research might extend the methodology to operation in positive mode and tackle analysis of cationic, zwitterionic and non‐ionic PFAS. It should also consider effects and necessary removal of solid phase material and co‐organics in complex PFAS samples, for example, blood [75] and AFFFs [3]. Capabilities for other chain lengths, including ‘ultrashorts’ (≤C4), and isomeric analysis should also be assessed. Finally, previous work warns that some PFAS are not easily ionised [9], such as secondary amine perfluorooctanyl sulphonamides {CF_3_(CF_2_)_X_SO_2_N(R)(R′) where R and R′ are alkyl groups} [76] and are typically assessed by GC–MS using electron‐ or chemical‐ionisation [67]. Future work should therefore assess if such species can be analysed using DPiMS.

## 4 Conclusions

There is no one analytical method that is appropriate for all PFAS concentrations, instead methods have been developed using a wide range of instruments to suit particular needs. This manuscript demonstrates that direct probe ionisation mass spectrometry DPiMS is a highly effective tool to add to the existing range of technologies for PFAS quantification at concentrations seen in environmental samples and PFAS remediation technologies. A linear response for PFOA over the range 2.36 μg L^−1^ to 5.20 mg L^−1^, *R* ^2^ > 0.99, was achieved with limit of detection of 0.828 μg L^−1^. Similar results were also observed for three other anionic PFAS (6:2 FTS, PFNA and PFOS). This performance is comparable with some existing and novel PFAS analysis techniques but also offers reduced analysis time, a simplified methodology and cheaper operational costs. The instrument offers near‐real time monitoring of PFAS destruction/removal technologies and reduces contamination errors from PTFE coatings and time dependant sorption to sample containers. The observed LOD and LOQ of the DPiMS for PFAS would be greater than those reported in this study for the analysis of samples in environmental matrices, but it is anticipated that using an established sample clean‐up and pre‐concentration method could alleviate these limitations. It is possible that the DPiMS would be most suitable for degradation studies in clean matrix. Further research could extend this methodology to shorter chain‐length PFAS, cationic and zwitterionic PFAS as well as more convoluted samples, such as groundwater and aqueous firefighting foams. The use of a triple quadrupole‐MS and further parametric optimisation could also improve limits of detection/quantification.

## Author Contributions

**Tim P. Sidnell:** conceptualization, methodology, writing – original draft, investigation, writing – review and editing. **Simone C. Mathias:** writing – review and editing, visualization, writing – original draft, methodology, investigation. **David P. Megson:** writing – review and editing. **Patrick J. Sears:** supervision, writing – review and editing, methodology, resources. **Madeleine J. Bussemaker:** conceptualization, funding acquisition, writing – review and editing, supervision, project administration, resources.

## Conflicts of Interest

The authors declare no conflicts of interest.

## Peer Review

The peer review history for this article is available at https://www.webofscience.com/api/gateway/wos/peer‐review/10.1002/rcm.10079.

## Supporting information


**Figure S1** 1) Sample slide, 2) Pipetting of 10 μL sample into sample well, 3) Placing of sample plate into DPiMS below the probe, 4) Insertion and withdrawal of probe in the sample, 5) Positioning of probe tip to align with DL and 6) Application of voltage to probe, ionising the sample which is drawn into the DL under vacuum
**Figure S2**: TIC (top) and integrated chromatograms for the DPiMS operating in scan mode (bottom left) and SIM mode (bottom right) (demonstrative values only
**Table S1**: Concentration ranges tested for the four PFAS in μM, mg L^−1^ and PPB
**Table S2**: Parameters affecting signal intensity of the DPiMS
**Figure S3**: Scan range TIC for 200 μM PFOA, three repeats shown. Note the PFOA peak at 413 and possible PFNA peak at Limit of detection

## Data Availability

The data that support the findings of this study are available from the corresponding author upon reasonable request.

## References

[1] R. C. Buck , J. Franklin , U. Berger , et al., “Perfluoroalkyl and Polyfluoroalkyl Substances in the Environment: Terminology, Classification, and Origins,” Integrated Environmental Assessment and Management 7, no. 4 (2011): 513–541, 10.1002/ieam.258. 21793199 PMC3214619

[2] Trier, DX , C Taxvig , AK Rosenmai , GA Pedersen , “PFAS in Paper and Board for Food Contact,” 2017 573. Nordic Council of Ministers; (2018), 10.6027/TN2017-573.

[3] J. L. Guelfo and C. P. Higgins , “Subsurface Transport Potential of Perfluoroalkyl Acids at Aqueous Film‐Forming Foam (AFFF)‐impacted Sites,” Environmental Science & Technology 47, no. 9 (2013): 4164–4171, 10.1021/es3048043. 23566120

[4] S. Fang , M. M. Plassmann , and I. T. Cousins , “Levels of Per‐ and Polyfluoroalkyl Substances (PFAS) in Ski Wax Products on the Market in 2019 Indicate no Changes in Formulation,” Environmental Science. Processes & Impacts 22, no. 11 (2020): 2142–2146, 10.1039/D0EM00357C. 33000820

[5] IPEN , “2019/Stockholm Convention COP‐9 White Paper,” The Global PFAS Problem: Fluorine‐Free Alternatives as Solutions, (2019), accessed May 7, 2024, www.ipen.org.

[6] Swedish Chemicals Agency (KEMI) 2015 Occurrence and Use of Highly Fluorinated Substances and Alternatives Report From a Government Assignment

[7] Smith JWN , B Beuthe M Dunk , “Environmental Fate and Effects of Poly‐and Perfluoroalkyl Substances (PFAS),” (2016).

[8] S. Rayne and K. Forest , “Perfluoroalkyl Sulfonic and Carboxylic Acids: A Critical Review of Physicochemical Properties, Levels and Patterns in Waters and Wastewaters, and Treatment Methods,” Journal of Environmental Science and Health Part a. 44, no. 12 (2009): 1145–1199, 10.1080/10934520903139811. 19847705

[9] J. W. Martin , K. Kannan , U. Berger , et al., “Peer Reviewed: Analytical Challenges Hamper Perfluoroalkyl Research,” Environmental Science & Technology 38, no. 13 (2004): 248A–255A, 10.1021/es0405528. 15296292

[10] C. Liu , K. Y. H. Gin , V. W. C. Chang , B. P. L. Goh , and M. Reinhard , “Novel Perspectives on the Bioaccumulation of PFCs ‐ The Concentration Dependency,” Environmental Science & Technology 45, no. 22 (2011): 9758–9764, 10.1021/es202078n. 21988464

[11] S. Kar , M. S. Sepúlveda , K. Roy , and J. Leszczynski , “Endocrine‐Disrupting Activity of Per‐ and Polyfluoroalkyl Substances: Exploring Combined Approaches of Ligand and Structure Based Modeling,” Chemosphere 184 (2017): 514–523, 10.1016/J.CHEMOSPHERE.2017.06.024. 28622647

[12] W. Wang , X. Hong , F. Zhao , J. Wu , and B. Wang , “The Effects of Perfluoroalkyl and Polyfluoroalkyl Substances on Female Fertility: A Systematic Review and Meta‐Analysis,” Environmental Research 216 (2023): 114718, 10.1016/J.ENVRES.2022.114718. 36334833

[13] E. Gorrochategui , E. Pérez‐Albaladejo , J. Casas , S. Lacorte , and C. Porte , “Perfluorinated Chemicals: Differential Toxicity, Inhibition of Aromatase Activity and Alteration of Cellular Lipids in Human Placental Cells,” Toxicology and Applied Pharmacology 277, no. 2 (2014): 124–130, 10.1016/j.taap.2014.03.012. 24680846

[14] L. Wilder , R. Worley , and P. Breysse , “Community Exposures to Per‐ and Polyfluoroalkyl Substances in Drinking Water: A National Issue ‐ ProQuest,” Journal of Environmental Heatlh 80, no. 2 (2017): 38–41, https://www.proquest.com/docview/1938071859/fulltextPDF/B631A3A8CF6545B2PQ/1?accountid=17256&sourcetype=Scholarly%20Journals.

[15] Stockholm Convention , “Press Releases|COP4 ‐ Geneva,” May 2009, Stockholm Convention, (2009), accessed May 7, 2024, https://chm.pops.int/Implementation/PublicAwareness/PressReleases/COP4Geneva,May2009/tabid/707/Default.aspx.

[16] Stockholm Convention , “Call for Information and Follow‐Up to the Ninth Meeting of the Conference of the Parties to the Stockholm Convention,” (2019), accessed May 7, 2024, https://chm.pops.int/theconvention/conferenceoftheparties/meetings/cop9/followuptocop9/tabid/8043/default.aspx.

[17] J. McDonough , J. Hurst , J. A. L. Miles , and T. Pancras , “Emerging Contaminants Handbook,” in *Emerging Contaminants Handbook* , 1st ed., eds. C. H. Bell , M. Gentile , E. Kalve , I. Ross , J. Horst , and S. Suthersan (CRC Press, 2019): 85–257, 10.1201/b22226/.

[18] C. Gallen , D. Drage , S. Kaserzon , et al., “Occurrence and Distribution of Brominated Flame Retardants and Perfluoroalkyl Substances in Australian Landfill Leachate and Biosolids,” Journal of Hazardous Materials 312 (2016): 55–64, 10.1016/j.jhazmat.2016.03.031. 27016666

[19] L. Ahrens , “Polyfluoroalkyl Compounds in the Aquatic Environment: A Review of Their Occurrence and Fate,” Journal of Environmental Monitoring 13, no. 1 (2011): 20–31, 10.1039/C0EM00373E. 21031178

[20] W. J. Backe , T. C. Day , and J. A. Field , “Zwitterionic, Cationic, and Anionic Fluorinated Chemicals in Aqueous Film Forming Foam Formulations and Groundwater From U.S. Military Bases by Nonaqueous Large‐Volume Injection HPLC‐MS/MS,” Environmental Science & Technology 47, no. 10 (2013): 5226–5234, 10.1021/es3034999. 23590254

[21] H. Chen , C. Zhang , J. Han , Y. Yu , and P. Zhang , “PFOS and PFOA in Influents, Effluents, and Biosolids of Chinese Wastewater Treatment Plants and Effluent‐Receiving Marine Environments,” Environmental Pollution 170 (2012): 26–31, 10.1016/j.envpol.2012.06.016. 22763327

[22] V. Mulabagal , L. Liu , J. Qi , C. Wilson , and J. S. Hayworth , “A Rapid UHPLC‐MS/MS Method for Simultaneous Quantitation of 23 Perfluoroalkyl Substances (PFAS) in Estuarine Water,” Talanta 190 (2018): 95–102, 10.1016/j.talanta.2018.07.053. 30172548

[23] DWI , “Drinking Water Inspectorate Guidance to Water Companies,” (2025).

[24] OECD , “Toward a New Comprehensive Global Database of Per‐ and Polyfluoroalkyl Substances (PFASs): Summary Report on Updating the OECD 2007 List of Per‐ and Polyfluoroalkyl Substances (PFASs),” (2018)

[25] S. K. Ostertag , B. A. Tague , M. M. Humphries , S. A. Tittlemier , and H. M. Chan , “Estimated Dietary Exposure to Fluorinated Compounds From Traditional Foods Among Inuit in Nunavut, Canada,” Chemosphere 75, no. 9 (2009): 1165–1172, 10.1016/j.chemosphere.2009.02.053. 19342075

[26] D. Megson , E. J. Reiner , K. J. Jobst , F. L. Dorman , M. Robson , and J. F. Focant , “A Review of the Determination of Persistent Organic Pollutants for Environmental Forensics Investigations,” Analytica Chimica Acta 941 (2016): 10–25, 10.1016/j.aca.2016.08.027. 27692373

[27] K. Gao , Y. Chen , Q. Xue , et al., “Trends and Perspectives in Per‐and Polyfluorinated Alkyl Substances (PFASs) Determination: Faster and Broader,” TrAC, Trends in Analytical Chemistry 133 (2020): 116114, 10.1016/j.trac.2020.116114.

[28] G. Munoz , S. Vo Duy , H. Budzinski , P. Labadie , J. Liu , and S. Sauvé , “Quantitative Analysis of Poly‐ and Perfluoroalkyl Compounds in Water Matrices Using High Resolution Mass Spectrometry: Optimization for a Laser Diode Thermal Desorption Method,” Analytica Chimica Acta 881 (2015): 98–106, 10.1016/j.aca.2015.04.015. 26041525

[29] E. F. Houtz , R. Sutton , J. S. Park , and M. Sedlak , “Poly‐ and Perfluoroalkyl Substances in Wastewater: Significance of Unknown Precursors, Manufacturing Shifts, and Likely AFFF Impacts,” Water Research 95 (2016): 142–149, 10.1016/j.watres.2016.02.055. 26990839

[30] T. Shende , G. Andaluri , and R. P. S. Suri , “Kinetic Model for Sonolytic Degradation of Non‐Volatile Surfactants: Perfluoroalkyl Substances,” Ultrasonics Sonochemistry 51 (2019): 359–368, 10.1016/j.ultsonch.2018.08.028. 30219351

[31] E. Ahmed , K. M. Mohibul Kabir , H. Wang , D. Xiao , J. Fletcher , and W. A. Donald , “Rapid Separation of Isomeric Perfluoroalkyl Substances by High‐Resolution Differential ion Mobility Mass Spectrometry,” Analytica Chimica Acta 1058 (2019): 127–135, 10.1016/j.aca.2019.01.038. 30851846

[32] S. Lath , E. R. Knight , D. A. Navarro , R. S. Kookana , and M. J. McLaughlin , “Sorption of PFOA Onto Different Laboratory Materials: Filter Membranes and Centrifuge Tubes,” Chemosphere 222 (2019): 671–678, 10.1016/j.chemosphere.2019.01.096. 30735967

[33] B. Chandramouli , J. P. Benskin , M. C. Hamilton , and J. R. Cosgrove , “Sorption of Per‐ and Polyfluoroalkyl Substances (PFASs) on Filter Media: Implications for Phase Partitioning Studies,” Environmental Toxicology and Chemistry 34, no. 1 (2015): 30–36, 10.1002/etc.2751. 25220773

[34] C. D. Vecitis , H. Park , J. Cheng , B. T. Mader , and M. R. Hoffmann , “Enhancement of Perfluorooctanoate and Perfluorooctanesulfonate Activity at Acoustic Cavitation Bubble Interfaces,” Journal of Physical Chemistry C 112, no. 43 (2008): 16850–16857, 10.1021/jp804050p.

[35] Lee, PJ , ET Bernier , GT Fujimoto , JC Shia , MS Young , AJ Di Gioia , “ACQUITY UPLC System Solution for Quantifying Trace Levels of Perfluorinated Compounds with an ACQUITY PFC Analysis Kit,” (2009).

[36] D. Martin , G. Munoz , S. Mejia‐Avendaño , et al., “Zwitterionic, Cationic, and Anionic Perfluoroalkyl and Polyfluoroalkyl Substances Integrated Into Total Oxidizable Precursor Assay of Contaminated Groundwater,” Talanta 195 (2019): 533–542, 10.1016/j.talanta.2018.11.093. 30625579

[37] J. Janda , K. Nödler , M. Scheurer , et al., “Closing the Gap – Inclusion of Ultrashort‐Chain Perfluoroalkyl Carboxylic Acids in the Total Oxidizable Precursor (TOP) Assay Protocol,” Environmental Science. Processes & Impacts 21, no. 11 (2019): 1926–1935, 10.1039/C9EM00169G. 31183483

[38] R. James Wood , T. Sidnell , I. Ross , J. McDonough , J. Lee , and M. J. Bussemaker , “Ultrasonic Degradation of Perfluorooctane Sulfonic Acid (PFOS) Correlated With Sonochemical and Sonoluminescence Characterisation,” Ultrasonics Sonochemistry 68 (2020): 105196, 10.1016/j.ultsonch.2020.105196. 32593965

[39] E. E. Ritter , M. E. Dickinson , J. P. Harron , et al., “PIGE as a Screening Tool for Per‐ and Polyfluorinated Substances in Papers and Textiles,” Nuclear Instruments and Methods in Physics Research B 407 (2017): 47–54, 10.1016/j.nimb.2017.05.052.

[40] A. E. Robel , K. Marshall , M. Dickinson , et al., “Closing the Mass Balance on Fluorine on Papers and Textiles,” Environmental Science & Technology 51, no. 16 (2017): 9022–9032, 10.1021/acs.est.7b02080. 28712295

[41] L. Schultes , G. F. Peaslee , J. D. Brockman , et al., “Total Fluorine Measurements in Food Packaging: How Do Current Methods Perform?,” Environmental Science & Technology Letters 6, no. 2 (2019): 73–78, 10.1021/acs.estlett.8b00700.

[42] C. Fang , J. Wu , Z. Sobhani , M. Al Amin , and Y. Tang , “Aggregated‐Fluorescent Detection of PFAS with a Simple Chip,” Analytical Methods 11, no. 2 (2019): 163–170, 10.1039/C8AY02382D.

[43] Cain, J , Z Powers , “Developing an Analytical Technique for PFAS in Water Using SPE and NMR,” Worcester Polytechnic Institute, (2020).

[44] K. Hiraoka , K. Nishidate , K. Mori , D. Asakawa , and S. Suzuki , “Development of Probe Electrospray Using a Solid Needle,” Rapid Communications in Mass Spectrometry 21, no. 18 (2007): 3139–3144, 10.1002/rcm.3201. 17708527

[45] K. Hiraoka , O. Ariyada , D. T. Usmanov , et al., “Probe Electrospray Ionization (PESI) and Its Modified Versions: Dipping PESI (dPESI), Sheath‐Flow PESI (sfPESI) and Adjustable sfPESI (Ad‐sfPESI),” Mass Spectrometry 9, no. 1 (2020): A0092, 10.5702/massspectrometry.A0092. 33299735 PMC7708747

[46] E. de Hoffman and V. Stroobant , *Mass Spectrometry Principles and Applications* , Third ed. (John Wiley & Sons, 2007).

[47] Monge, ME , FM Fernández , “An Introduction to Ambient Ionization Mass Spectrometry,” New Developments in Mass Spectrometry, (2014),2015‐January(2):1–22, 10.1039/9781782628026-00001.

[48] R. V. Emmons , W. Fatigante , A. A. Olomukoro , B. Musselman , and E. Gionfriddo , “Rapid Screening and Quantification of PFAS Enabled by SPME‐DART‐MS,” Journal of the American Society for Mass Spectrometry 34, no. 9 (2023): 1890–1897, 10.1021/jasms.3c00088. 37260314

[49] J. Deng , Y. Yang , L. Fang , L. Lin , H. Zhou , and T. Luan , “Coupling Solid‐Phase Microextraction With Ambient Mass Spectrometry Using Surface Coated Wooden‐Tip Probe for Rapid Analysis of Ultra Trace Perfluorinated Compounds in Complex Samples,” Analytical Chemistry 86, no. 22 (2014): 11159–11166, 10.1021/ac5034177. 25354323

[50] P. Suwannakot , F. Lisi , E. Ahmed , et al., “Metal‐Organic Framework‐Enhanced Solid‐Phase Microextraction Mass Spectrometry for the Direct and Rapid Detection of Perfluorooctanoic Acid in Environmental Water Samples,” Analytical Chemistry 92, no. 10 (2020): 6900–6908, 10.1021/acs.analchem.9b05524. 32329336

[51] J. Deng , Y. Yang , M. Xu , et al., “Surface‐Coated Probe Nanoelectrospray Ionization Mass Spectrometry for Analysis of Target Compounds in Individual Small Organisms,” Analytical Chemistry 87, no. 19 (2015): 9923–9930, 10.1021/acs.analchem.5b03110. 26360344

[52] Shimadzu , “Instruction Manual ‐ Direct Probe Ionization Mass Spectrometer (DPiMS‐2020)”.

[53] M. F. Rahman , S. Peldszus , and W. B. Anderson , “Behaviour and Fate of Perfluoroalkyl and Polyfluoroalkyl Substances (PFASs) in Drinking Water Treatment: A Review,” Water Research 50 (2014): 318–340, 10.1016/j.watres.2013.10.045. 24216232

[54] X. Trier , K. Granby , and J. H. Christensen , “Polyfluorinated Surfactants (PFS) in Paper and Board Coatings for Food Packaging,” Environmental Science and Pollution Research 18, no. 7 (2011): 1108–1120, 10.1007/s11356-010-0439-3. 21327544

[55] Z. U. Zango , B. Ethiraj , F. S. Al‐Mubaddel , et al., “An Overview on Human Exposure, Toxicity, Solid‐Phase Microextraction and Adsorptive Removal of Perfluoroalkyl Carboxylic Acids (PFCAs) From Water Matrices,” Environmental Research 231 (2023): 116102, 10.1016/J.ENVRES.2023.116102. 37196688

[56] M. K. Björnsdotter , W. F. Hartz , R. Kallenborn , et al., “Levels and Seasonal Trends of C1‐C4 Perfluoroalkyl Acids and the Discovery of Trifluoromethane Sulfonic Acid in Surface Snow in the Arctic,” Environmental Science & Technology 55, no. 23 (2021): 15853–15861, 10.1021/ACS.EST.1C04776/ASSET/IMAGES/LARGE/ES1C04776_0004.JPEG. 34779623 PMC8655978

[57] J. B. Fenn , M. Mann , C. K. Meng , S. F. Wong , and C. M. Whitehouse , “Electrospray Ionization–Principles and Practice,” Mass Spectrometry Reviews 9, no. 1 (1990): 37–70, 10.1002/mas.1280090103.

[58] G. A. Valaskovic , J. P. Murphy , and M. S. Lee , “Automated Orthogonal Control System for Electrospray Ionization,” Journal of the American Society for Mass Spectrometry 15, no. 8 (2004): 1201–1215, 10.1016/j.jasms.2004.04.033. 15276167

[59] C. A. Huset and M. K. Barry , “Quantitative Determination of Perfluoroalkyl Substances (PFAS) in Soil, Water, and Home Garden Produce,” Methods 5 (2018): 697–704, 10.1016/j.mex.2018.06.017. PMC6039355 29998069

[60] R. A. Brase and D. C. Spink , “Enhanced Sensitivity for the Analysis of Perfluoroethercarboxylic Acids Using LC‐ESI‐MS/MS: Effects of Probe Position, Mobile Phase Additive, and Capillary Voltage,” Journal of the American Society for Mass Spectrometry 31, no. 10 (2020): 2124–2132, 10.1021/jasms.0c00244. 32794713

[61] Organtini, K , S Oehrle , K Rosnack . “An Alternative Ionization Technique for LC‐MS/MS Analysis of Perfluoroalkyl Substances (PFAS) in Environmental Samples,” (2021).

[62] J. Yu , A. Nickerson , Y. Li , Y. Fang , and T. J. Strathmann , “Fate of Per‐ and Polyfluoroalkyl Substances (PFAS) During Hydrothermal Liquefaction of Municipal Wastewater Treatment Sludge,” Environmental Sciences 6, no. 5 (2020): 1388–1399, 10.1039/C9EW01139K.

[63] United States Environmental Protection Agency (EPA) , “PFAS National Primary Drinking Water Regulation,” (2024).

[64] E. Schepens , S. Inman , B. J. McCullough , and C. Hopley , “Rapid Confirmation and Quantitation of Drugs‐of‐Abuse in Oral Fluid Using a Low Cost, Small Footprint Mass Spectrometer,” Forensic Chemistry 4 (2017): 75–81, 10.1016/j.forc.2017.03.002.

[65] B. J. Mccullough , K. Patel , R. Francis , et al., “Atmospheric Solids Analysis Probe Coupled to a Portable Mass Spectrometer for Rapid Identification of Bulk Drug Seizures,” Journal of the American Society for Mass Spectrometry 31, no. 2 (2020): 386–393, 10.1021/jasms.9b00020. 32031401

[66] C. Bell , M. Gentile , E. Kalve , I. Ross , J. Horst , and S. Suthersan , “Emerging Contaminants Handbook,” in *Emerging Contaminants Handbook* , ed. F. L. Boca Raton (CRC Press, 2019): 85–257.

[67] D. W. Kuehl and B. Rozynov , “Chromatographic and Mass Spectral Studies of Perfluorooctanesulfonate and Three Perfluorooctanesulfonamides,” Rapid Communications in Mass Spectrometry 17, no. 20 (2003): 2364–2369, 10.1002/rcm.1181. 14558140

[68] H. Cao , W. Zhang , C. Wang , and Y. Liang , “Sonochemical Degradation of Poly‐ and Perfluoroalkyl Substances – A Review,” Ultrasonics Sonochemistry 69 (2020): 105245, 10.1016/j.ultsonch.2020.105245. 32702636

[69] N. E. Pica , J. Funkhouser , Y. Yin , et al., “Electrochemical Oxidation of Hexafluoropropylene Oxide Dimer Acid (GenX): Mechanistic Insights and Efficient Treatment Train With Nanofiltration,” Environmental Science & Technology 53, no. 21 (2019): 12602–12609, 10.1021/acs.est.9b03171. 31599577

[70] A. J. Lewis , T. Joyce , M. Hadaya , et al., “Rapid Degradation of PFAS in Aqueous Solutions by Reverse Vortex Flow Gliding arc Plasma,” Environmental Sciences 6, no. 4 (2020): 1044–1057, 10.1039/C9EW01050E.

[71] J. Horst , J. McDonough , I. Ross , et al., “Water Treatment Technologies for PFAS: The Next Generation,” Groundwater Monitoring and Remediation 38, no. 2 (2018): 13–23, 10.1111/gwmr.12281.

[72] Ross I J Hurst E Kalve E Houtz T Pancras “Remediation of Poly‐ and Perfluoro Alkyl Substances: New Remediation Technologies for Emerging Challenges,” (2016)

[73] N. Merino , Y. Qu , R. A. Deeb , E. L. Hawley , M. R. Hoffmann , and S. Mahendra , “Degradation and Removal Methods for Perfluoroalkyl and Polyfluoroalkyl Substances in Water,” Environmental Engineering Science 33, no. 9 (2016): 615–649, 10.1089/ees.2016.0233.

[74] S. Woodard , J. Berry , and B. Newman , “Ion Exchange Resin for PFAS Removal and Pilot Test Comparison to GAC,” Remediation Journal 27, no. 3 (2017): 19–27, 10.1002/rem.21515.

[75] J. Belisle and D. F. Hagen , “A Method for the Determination of Perfluorooctanoic Acid in Blood and Other Biological Samples,” Analytical Biochemistry 101, no. 2 (1980): 369–376, 10.1016/0003-2697(80)90202-X. 7362033

[76] J. W. Martin , D. C. G. Muir , C. A. Moody , et al., “Collection of Airborne Fluorinated Organics and Analysis by Gas Chromatography/Chemical Ionization Mass Spectrometry,” Analytical Chemistry 74, no. 3 (2002): 584–590, 10.1021/ac015630d. 11842814

