# Supplementary material for: Direct Probe Ionisation Mass Spectrometry for Rapid and Accurate Determination of Perfluoroalkyl and Polyfluoroalkyl Substance Concentrations
Source: Rapid Commun Mass Spectrom. 2025 Jun 11;39(18):e10079. doi: 10.1002/rcm.10079 (PMC12152711; doi:10.1002/rcm.10079)
Supplement: Supplementary file 1 — Figure S1 1) Sample slide, 2) Pipetting of 10 μL sample into sample well, 3) Placing of sample plate into DPiMS below the probe, 4) Insertion and withdrawal of probe in the sample, 5) Positioning of probe tip to align with DL and 6) Application of voltage to probe, ionising the sample which is drawn into the DL under vacuum Figure S2: TIC (top) and integrated chromatograms for the DPiMS operating in scan mode (bottom left) and SIM mode (bottom right) (demonstrative values only Table S1: Concentration ranges tested for the four PFAS in μM, mg L−1 and PPB Table S2: Parameters affecting signal intensity of the DPiMS Figure S3: Scan range TIC for 200 μM PFOA, three repeats shown. Note the PFOA peak at 413 and possible PFNA peak at Limit of detection [file RCM-39-e10079-s001.docx]

**Supplementary Information: Direct Probe ionisation Mass Spectrometry for rapid and accurate determination of Per- and Polyfluoroalkyl Substance concentrations**

*Tim P. Sidnell ^a^, Simone C. Mathias ^a^, David P. Megson ^b^_,_ Patrick J. Sears ^a^* and Madeleine J. Bussemaker ^a^*

*^a^ School of Chemistry and Chemical Engineering, University of Surrey, Guildford, Surrey, United Kingdom, GU2 7XH*

*^b^ Ecology & Environment Research Centre, Manchester Metropolitan University, John Dalton Building, Manchester, United Kingdom M1 5GD*

*** [*p.sears@surrey.ac.uk*](mailto:p.sears@surrey.ac.uk)

Contents

[S1. DPiMS operation 2](#_Toc186706965)

[S2. Dilution series preparation 4](#_Toc186706966)

[S3. Parameters assessment choice and optimisation procedure 6](#_Toc186706967)

[S4. Internal standard selection 8](#_Toc186706968)

[Supplementary Information References 10](#_Toc186706969)

List of figures

[Figure S1: 1) Sample slide, 2) Pipetting of 10 μl sample into sample well, 3) Placing of sample plate into DPiMS below the probe, 4) Insertion and withdrawal of probe in the sample, 5) Positioning of probe tip to align with DL and 6) Application of voltage to probe, ionising the sample which is drawn into the DL under vacuum 3](#_Toc174351721)

[Figure S2: TIC (top) and integrated chromatograms for the DPiMS operating in scan mode (bottom left) and SIM mode (bottom right) (demonstrative values only 4](#_Toc174351722)

[Figure S3: Scan range TIC for 200 μM PFOA, three repeats shown. Note the PFOA peak at 413 and possible PFNA peak at 463 9](#_Toc174351723)

[Figure S4: Plots of PFOS intensity (AU) vs PFOS concentration (µM) at scan ranges of m/z 50-550 (orange triangles) and m/z 490-510 (blue circles). 10](#_Toc174351724)

List of tables

[Table S1: Concentration ranges tested for the four PFAS in μM, mg L^-1^ and PPB 5](#_Toc190336305)

[Table S2: Parameters affecting signal intensity of the DPiMS 7](#_Toc190336306)

# DPiMS operation

A 10 µl sample is pipetted into a well on the polypropylene sample slide, which is placed below the sampling needle (probe), within a closed container (Figure S1 1-3) [1]. The probe then repeatedly lowers into and rises out of the sample well (Figure S1 4). When risen, the probe aligns the sample droplet adjacent to a de-solvation line (DL) (Figure S1 5) before an electrical charge is applied to the droplet, causing the sample to undergo electrospray ionisation at atmospheric pressure (Figure A.6). Ions align at the surface of the liquid droplet and are then drawn under vacuum through the DL. The DL is heated, which allows excess solvent to evaporate away from the droplets, leaving behind the ions to be detected Figure S1 6. The ion fragments are then separated based on their mass to charge ratios (m/z values) by a quadrupole mass filter, before being detected by a secondary electron multiplier with an enabled conversion dynode. Note that a DPiMS with a triple quadrupole system is also available. This process then repeats, with the probe taking several picolitre-sized samples from the slide (around 40 over the course of a minute, depending on the settings used). Once the signals are detected, they are amplified before being analysed in an associated software [1]. The DPiMS offers three operational modes; 1) Scan mode, which detects any ions above the LOD, within a specified *m/z* range 2) Single Ion Monitoring (SIM) mode, which detects prespecified m/z values and 3) Profile mode, which shows the Total Ion Chromatogram (TIC) as intensity data vs *m/z*, as opposed to the SIM centroid bar graph [1].

Figure S1: 1) Sample slide, 2) Pipetting of 10 μl sample into sample well, 3) Placing of sample plate into DPiMS below the probe, 4) Insertion and withdrawal of probe in the sample, 5) Positioning of probe tip to align with DL and 6) Application of voltage to probe, ionising the sample which is drawn into the DL under vacuum

Figure S2: TIC (top) and integrated chromatograms for the DPiMS operating in scan mode (bottom left) and SIM mode (bottom right) (demonstrative values only

# Dilution series preparation

The PFAS powders used were reluctant to dissolve at 200 μM, despite this concentration being far below the aqueous saturation values of PFOA and PFOS at 20°C (8.21 mM and 1.03 mM, respectively) [2]. Note that no consistent data could be found on the saturation values for PFNA and 6:2 FTS. All 200 μM solutions were therefore magnetically stirred at 1,000 RPM and heated to 40°C for approximately one hour, such that visible dissolution was achieved. PFAS are thermally stable, by design, and 40°C is far below temperatures required to break them down (e.g. ≥870°C [3]). The PFAS remained in solution after the mixing/heating was stopped and the solutions were then diluted and combined as necessary. The dilution series were made in glass containers, which absorbs the least PFAS of several materials tested previously [4]. The dilution series were refrigerated at 4°C and analysed within 6 hours to minimise time-dependant container-sorption losses [5], [4], [6]. 5 minutes prior to analysis, the analyte series were manufactured in 2 ml polypropylene Eppendorf vials, using 1 ml polypropylene pipette tips. The analytes comprised 1 ml ethanol, 0.5 ml aqueous PFAS and 0.5 ml Milli-Q water or aqueous internal standard (see section 2.6). To ionise samples, the DPiMS manufacturer recommends using 30-70% v/v organic solvent [1]. Hence, 50% organic solvent was used here, though this value is not optimised. The short retention time within the Eppendorf vials and use of ethanol limited the sorption of the PFAS to the polypropylene walls [7]. 10 µl analyte samples were then pipetted onto the DPiMS polypropylene sample slide using polypropylene positive displacement pipette tips and placed below the probe (Figure S1).

Table S1: Concentration ranges tested for the four PFAS in μM, mg L^-1^ and PPB

| **PFAS**  [RMM (Da)] | **Concentration range assessed** | | | | | |
| --- | --- | --- | --- | --- | --- | --- |
|  | **μM** | | **mg L^-1^** | | **PPB** | |
| **PFOA**  [414.07] | **Series 1.1** | **Series 2** | **Series 1.1** | **Series 2** | **Series 1.1** | **Series 2** |
|  | 2.000 x 10^-3^  2.000 x 10^-2^  2.000 x 10^-1^  2.000 x 10^0^  2.000 x 10^1^  2.000 x 10^2^ | 5.000 x 10^-4^  5.000 x 10^-3^  5.000 x 10^-2^  5.000 x 10^-1^  5.000 x 10^0^  5.000 x 10^1^ | 8.281 x 10^-4^  8.281 x 10^-3^  8.281 x 10^-2^  8.281 x 10^-1^  8.281 x 10^0^  8.281 x 10^1^ | 2.070 x 10^-4^  2.070 x 10^-3^  2.070 x 10^-2^  2.070 x 10^-1^  2.070 x 10^0^  2.070 x 10^1^ | 0.8281  8.281  82.81  828.1  8,281  82,810 | 0.2070  2.070  20.70  207.0  2,070  20,700 |
| **6:2 FTS**  [428.17] | Not assessed | 5.000 x 10^-4^  5.000 x 10^-3^  5.000 x 10^-2^  5.000 x 10^-1^  5.000 x 10^0^  5.000 x 10^1^ | Not assessed | 2.140 x 10^-4^  2.140 x 10^-3^  2.140 x 10^-2^  2.140 x 10^-1^  2.140 x 10^0^  2.140 x 10^1^ | Not assessed | 0.2141  2.141  21.41  214.1  2,141  21,410 |
| **PFNA**  [464.08] | **Series 1.2** | **Series 2** | **Series 1.2** | **Series 2** | **Series 1.2** | **Series 2** |
|  | 2.000 x 10^-3^  2.000 x 10^-2^  2.000 x 10^-1^  2.000 x 10^0^  2.000 x 10^1^  2.000 x 10^2^ | 5.000 x 10^-4^  5.000 x 10^-3^  5.000 x 10^-2^  5.000 x 10^-1^  5.000 x 10^0^  5.000 x 10^1^ | 9.282 x 10^-4^  9.282 x 10^-3^  9.282x 10^-2^  9.282 x 10^-1^  9.282 x 10^0^  9.282 x 10^1^ | 2.320 x 10^-4^  2.320 x 10^-3^  2.320 x 10^-2^  2.320 x 10^-1^  2.320 x 10^0^  2.320 x 10^1^ | 0.9281  9.282  92.82  928.2  9,282  92,820 | 0.2320  2.320  23.20  232.0  2,320  23,200 |
| **K-PFOS**  [538.22] | **Series 1.3** | **Series 2** | **Series 1.3** | **Series 2** | **Series 1.3** | **Series 2** |
|  | 2.000 x 10^-3^  2.000 x 10^-2^  2.000 x 10^-1^  2.000 x 10^0^  2.000 x 10^1^  2.000 x 10^2^ | 5.000 x 10^-4^  5.000 x 10^-3^  5.000 x 10^-2^  5.000 x 10^-1^  5.000 x 10^0^  5.000 x 10^1^ | 1.077 x 10^-3^  1.077 x 10^-2^  1.077 x 10^-1^  1.077 x 10^0^  1.077 x 10^1^  1.077 x 10^2^ | 2.690 x 10^-4^  2.690 x 10^-3^  2.690 x 10^-2^  2.690 x 10^-1^  2.690 x 10^0^  2.690 x 10^1^ | 1.076  10.76  107.6  1,076  10,764  107,650 | 0.2691  2.691  26.91  269.1  2,691  26,910 |

# Parameters assessment choice and optimisation procedure

The parameters affecting the intensity reading of the DPiMS can be broken down into three categories; sample chemical properties, probe ionisation parameters and mass spectrometer parameters. Table B lists the most relevant of each of these parameter types, which were identified from the Shimadzu DPiMS-2020 user manual [1] and the authors’ experience of operating the device. Tick marks (🗸) indicate which parameters considered in this work and the ranges over which the parameters were tested are given in brackets. Sample concentration was the main indicator of device performance, indicating the LOD/Qs of the device for the different PFAS tested. Hence, this was the initial parameter investigated using calibration series for each PFAS tested (PFOA, PFNA and PFOS) from 2 nM to 200 μM. LOD/LOQs could then be enhanced through parameter optimisation on a on a *one factor at a time* basis [8]. The monitoring mode greatly effects sensitivity, since scan mode allows detection of a greater number of compounds, with reduced sensitivity compared to SIM mode [5], [9], [10]. For this reason, most investigations in this work focused on obtaining low-level detection of and quantification of samples using SIM mode. Hence, this was one of the initial parameters investigated. Sample slide and probe cleaning were assessed since initial tests revealed some carryover between samples, determined by successively higher readings seen in repeats of the same samples. The ionising solvent choice was not investigated, although results discussed in section 3 suggest that acetone is not an appropriate solvent to use, while ethanol worked consistently in this study. Once a reliable cleaning methodology and monitoring mode were revealed, ionising voltage and DL heating temperature were optimised, in that order, using the peak intensity of PFOA seen at 413 Th, representing the mass of the PFOA anion formed when a terminal hydrogen is removed during dissolution of the PFOA salt. The DL heating temperature determines how much solvent is evaporated from the ionised droplets and, in turn, affects the sensitivity of the MS to incoming ions. The ions will not reach the detector if the solvent evaporates too quickly but will not be detected if insufficient evaporation occurs. The ionising voltage is critical in forming charged species [11] since too high an ionising voltage may result in compounds being decomposed and hence not detected at the expected *m/z* value, while too low a voltage will not allow sufficient ionisation. Hence, both parameters are critical to find the optimum detection conditions. The effects of probe ionisation depth have been shown to be most significant only at the optimised ionisation voltage [11]. Hence, probe immersion and ionisation depth were not assessed here, since they are considered secondary parameters.

Table S2: Parameters affecting signal intensity of the DPiMS

| **Sample Properties** | | **Ionisation Parameters** | | **MS Parameters** | |
| --- | --- | --- | --- | --- | --- |
| Sample slide cleaning between samples (Yes/No) | 🗸 | DL temperature  (100 - 250°C) | 🗸 | Conducting voltage |  |
| Sample concentration  (2 nM – 200 µM) | 🗸 | Ionisation voltage  (-2.45 – -1.75 kV) | 🗸 | Monitoring mode  (SIM/Scan) | 🗸 |
| Sample make up  (Single/Multiple PFAS) | 🗸 | Number of measuring events |  |  |  |
| Solvent choice |  | Probe cleaning between samples (Yes/No) | 🗸 |  |  |
| V/V Solvent % |  | Depth of probe immersion |  |  |  |
| Viscosity |  | Depth of probe ionisation |  |  |  |

# Internal standard selection

For quantifying unknown samples, an internal standard (IS) must be added for which the plot of intensity vs ratio of IS: analyte concentration is known. During electrospray ionisation (ESI), co-PFAS with similar ionic properties can enhance analyte PFAS signals and increase LODs/Qs [5]. In scan mode, an IS with a similar *m/z* value to the analyte minimises the necessary scan range and improves sensitivity/run time. Some PFAS destructive technologies can generate shorter-chain PFAS by removing CF_2_ or CH_2_ units in the perfluoro-chain, [12]–[21], however, chain lengthening has never been observed. Therefore, for monitoring such reactions, samples require an IS with a longer perfluoro chain than the analyte. PFNA was selected as the IS for PFOA, since it has an anionic nature, an m/z value close to PFOA’s, (*m/z* 463 vs 413), near-identical structure and a longer perfluoro chain (C9 vs C8). PFOA intensity at *m/z* 413 was 1.37 x10^6^ (±1.88 x10^5^) AU, whereas PFNA intensity (*m/z* of 463) was 7.17 x10^3^ (±1.14 x10^3^) AU. Background noise was 4.365 x10^3^ (±5.74 x10^2^) AU, giving a PFNA signal to noise ratio below 3.000 (1.643). Hence, there were not statistically significant PFNA quantities in the PFOA. Any presence of PFOA in the internal standard was accounted for during background subtraction.

Figure S3: Scan range TIC for 200 μM PFOA, three repeats shown. Note the PFOA peak at 413 and possible PFNA peak at Limit of detection

# Supplementary Information References

[1] Shimadzu, “Direct Probe Ionization Mass Spectrometer (DPiMS-2020) Instruction Manual Shimadzu, 225-32571-02,” 2020.

[2] J. Alexander *et al.*, “Perfluorooctane sulfonate (PFOS), perfluorooctanoic acid (PFOA) and their salts Scientific Opinion of the Panel on Contaminants in the Food chain,” *EFSA J.*, vol. 6, no. 7, pp. 1–131, 2008.

[3] K. Aleksandrov *et al.*, “Waste incineration of Polytetrafluoroethylene (PTFE) to evaluate potential formation of per- and Poly-Fluorinated Alkyl Substances (PFAS) in flue gas,” *Chemosphere*, vol. 226, pp. 898–906, 2019.

[4] S. Lath, E. R. Knight, D. A. Navarro, R. S. Kookana, and M. J. McLaughlin, “Sorption of PFOA onto different laboratory materials: Filter membranes and centrifuge tubes,” *Chemosphere*, vol. 222, pp. 671–678, 2019.

[5] J. W. Martin *et al.*, “Peer Reviewed: Analytical Challenges Hamper Perfluoroalkyl Research,” *Environ. Sci. Technol.*, vol. 38, no. 13, pp. 248A-255A, 2004.

[6] B. Chandramouli, J. P. Benskin, M. C. Hamilton, and J. R. Cosgrove, “Sorption of per- and polyfluoroalkyl substances (PFASs) on filter media: Implications for phase partitioning studies,” *Environ. Toxicol. Chem.*, vol. 34, no. 1, pp. 30–36, 2015.

[7] N. Bolan *et al.*, “Remediation of poly- and perfluoroalkyl substances (PFAS) contaminated soils – To mobilize or to immobilize or to degrade?,” *J. Hazard. Mater.*, vol. 401, no. September 2020, 2021.

[8] G. Munoz, S. Vo Duy, H. Budzinski, P. Labadie, J. Liu, and S. Sauvé, “Quantitative analysis of poly- and perfluoroalkyl compounds in water matrices using high resolution mass spectrometry: Optimization for a laser diode thermal desorption method,” *Anal. Chim. Acta*, vol. 881, pp. 98–106, 2015.

[9] V. Mulabagal, L. Liu, J. Qi, C. Wilson, and J. S. Hayworth, “A rapid UHPLC-MS/MS method for simultaneous quantitation of 23 perfluoroalkyl substances (PFAS) in estuarine water,” *Talanta*, vol. 190, no. July, pp. 95–102, 2018.

[10] D. Panda, V. Sethu, and S. Manickam, “Kinetics and mechanism of low-frequency ultrasound driven elimination of trace level aqueous perfluorooctanesulfonic acid and perfluorooctanoic acid,” *Chem. Eng. Process. - Process Intensif.*, vol. 142, p. 107542, 2019.

[11] R. A. Brase and D. C. Spink, “Enhanced Sensitivity for the Analysis of Perfluoroethercarboxylic Acids Using LC-ESI-MS/MS: Effects of Probe Position, Mobile Phase Additive, and Capillary Voltage,” *J. Am. Soc. Mass Spectrom.*, vol. 31, no. 10, pp. 2124–2132, 2020.

[12] H. Moriwaki, Y. Takagi, M. Tanaka, K. Tsuruho, K. Okitsu, and Y. Maeda, “Sonochemical decomposition of perfluorooctane sulfonate and perfluorooctanoic acid,” *Environ. Sci. Technol.*, vol. 39, no. 9, pp. 3388–3392, 2005.

[13] R. James Wood, T. Sidnell, I. Ross, J. McDonough, J. Lee, and M. J. Bussemaker, “Ultrasonic degradation of perfluorooctane sulfonic acid (PFOS) correlated with sonochemical and sonoluminescence characterisation,” *Ultrason. Sonochem.*, vol. 68, no. August 2019, p. 105196, 2020.

[14] Y. C. Lee, S. L. Lo, J. Kuo, and Y. L. Lin, “Persulfate oxidation of perfluorooctanoic acid under the temperatures of 20-40°C,” *Chem. Eng. J.*, vol. 198–199, pp. 27–32, 2012.

[15] T. A. Bruton and D. L. Sedlak, “Treatment of Aqueous Film-Forming Foam by Heat-Activated Persulfate under Conditions Representative of in Situ Chemical Oxidation,” *Environ. Sci. Technol.*, vol. 51, no. 23, pp. 13878–13885, 2017.

[16] H. Hori *et al.*, “Decomposition of environmentally persistent perfluorooctanoic acid in water by photochemical approaches,” *Environ. Sci. Technol.*, vol. 38, no. 22, pp. 6118–6124, 2004.

[17] H. Hori *et al.*, “Efficient decomposition of environmentally persistent perfluorocarboxylic acids by use of persulfate as a photochemical oxidant,” *Environ. Sci. Technol.*, vol. 39, no. 7, pp. 2383–2388, 2005.

[18] Y. Wang, P. Zhang, G. Pan, and H. Chen, “Ferric ion mediated photochemical decomposition of perfluorooctanoic acid (PFOA) by 254 nm UV light,” *J. Hazard. Mater.*, vol. 160, no. 1, pp. 181–186, 2008.

[19] Z. Song, H. Tang, N. Wang, and L. Zhu, “Reductive defluorination of perfluorooctanoic acid by hydrated electrons in a sulfite-mediated UV photochemical system,” *J. Hazard. Mater.*, vol. 262, pp. 332–338, 2013.

[20] R. K. products from perfluorinated alkyl substances (PFAS) degradation in a plasma-based water treatment process Singh, S. Fernando, S. Fakouri Baygi, N. Multari, S. Mededovic Thagard, and T. M. Holsen, “Breakdown products from perfluorinated alkyl substances (PFAS) degradation in a plasma-based water treatment process,” *Environ. Sci. Technol.*, vol. 53, p. acs.est.8b07031, 2019.

[21] G. R. Stratton, F. Dai, C. L. Bellona, T. M. Holsen, E. R. V. Dickenson, and S. Mededovic Thagard, “Plasma-Based Water Treatment: Efficient Transformation of Perfluoroalkyl Substances in Prepared Solutions and Contaminated Groundwater,” *Environ. Sci. Technol.*, vol. 51, no. 3, pp. 1643–1648, 2017.
